# Genome-wide characterization of *vibrio* phage ϕpp2 with unique arrangements of the *mob*-like genes

**DOI:** 10.1186/1471-2164-13-224

**Published:** 2012-06-07

**Authors:** Ying-Rong Lin, Chan-Shing Lin

**Affiliations:** 1 Department of Marine Biotechnology and Resources, Asia-Pacific Ocean Research Center, National Sun Yat-sen University, Kaohsiung, 80424, Taiwan; 2 Agricultural Biotechnology Center, National Chung-Hsing University, Taichung, 402, Taiwan

**Keywords:** Homing endonuclease, T4-like phage, *Vibrio parahaemolyticus*

## Abstract

**Background:**

*Vibrio parahaemolyticus* is associated with gastroenteritis, wound infections, and septicemia in human and animals. Phages can control the population of the pathogen. So far, the only one reported genome among giant vibriophages is KVP40: 244,835 bp with 26% coding regions that have T4 homologs. Putative homing endonucleases (HE) were found in *Vibrio* phage KVP40 bearing one *seg*D and *Vibrio cholerae* phage ICP1 carrying one *mob*C/E and one *seg*G.

**Results:**

A newly isolated *Vibrio* phage ϕpp2, which was specific to the hosts of *V. parahaemolyticus* and *V. alginolyticus*, featured a long nonenveloped head of ~90 × 150 nm and tail of ~110 nm. The phage can survive at 50°C for more than one hour. The genome of the phage ϕpp2 was sequenced to be 246,421 bp, which is 1587 bp larger than KVP40. 383 protein-encoding genes (PEGs) and 30 tRNAs were found in the phage ϕpp2. Between the genomes of ϕpp2 and KVP40, 254 genes including 29 PEGs for viral structure were of high similarity, whereas 17 PEGs of KVP40 and 21 PEGs of ϕpp2 were unmatched. In both genomes, the capsid and tail genes have been identified, as well as the extensive representation of the DNA replication, recombination, and repair enzymes. In addition to the three giant indels of 1098, 1143 and 3330 nt, ϕpp2 possessed unique proteins involved in potassium channel, gp2 (DNA end protector), tRNA nucleotidyltransferase, and mob-type HEs, which were not reported in KVP40. The ϕpp2 PEG274, with strong promoters and translational initiation, was identified to be a *mob*E type, flanked by NrdA and NrdB/C homologs. Coincidently, several pairs of HE-flanking homologs with empty center were found in the phages of *Vibrio* phages ϕpp2 and KVP40, as well as in *Aeromonas* phages (Aeh1 and Ae65), and cyanophage P-SSM2.

**Conclusions:**

*Vibrio* phage ϕpp2 was characterized by morphology, growth, and genomics with three giant indels and different types of HEs. The gene analysis on the required elements for transcription and translation suggested that the ϕpp2 PEG274 was an active *mob*E gene. The phage was signified to be a new species of T4-related, differing from KVP40.

## Background

*Vibrio parahaemolyticus* is a halophilic gram-negative bacterium that is widely distributed in coastal waters worldwide and is associated with gastroenteritis, wound infections, and septicemia [1]. Since the first report of Fujino *et al.*[2], numerous investigations of *V. parahaemolyticus* have been performed using stools of patients and diseased fish. The halophile has been found seasonally in sea water of the continental United States, Germany, the Far East, and Hawaii [3-6]. *V. parahaemolyticus* infections are frequently reported to occur due to the consumption of undercooked raw shellfish or direct contact with estuarine waters. In Asia, many recent infections have been caused by serotype O3:K6 of *V. parahaemolyticus*[7].

The phages can control the population of the pathogen. Among the giant T4-like phages that are specific to *V. parahaemolyticus,* the vibriophage KVP40 is the only strain for which the genome has been determined [8]. The size of the KVP40 genome is 244,835 bp with an overall G + C content of 42.6%. It contains 381 putative protein-encoding genes (PEG), 30 tRNAs, 33 late promoters, and 57 rho-independent terminators. The genome sequence and organization of KVP40 show a degree of conservation with phage T4. While 65% of the PEGs were unique to KVP40, 99 out of the total 381 putative coding regions have homologs in the T4 genome, which includes DNA replication, recombination, and repair enzymes as well as the viral capsid and tail structural genes. KVP40 lacks enzymes involved in DNA degradation, cytosine modification and group I introns, and it probably utilizes NAD salvage pathway that is unique among bacteriophages [8].

Phages can prompt gene recombination via homing endonucleases (HEs). In genome analyses, putative homing endonucleases (HEs) were found in *Vibrio parahaemolyticus* phages KVP40 and *Vibrio cholerae* ICP1 [8,9]. Homing endonucleases might act as possible mediators for the diversity among bacteriophage genomes by the acquisition of a novel DNA to create a new species of phage. Although more than 30 T4-related genomes have been published so far, no other known phage genome comes close to encoding the 15 homing endonucleases in T4 phage [10-12]. Intron homing [13] and intronless homing [14,15] endonucleases both utilize homologous recombination between phages to transfer the genetic elements from the HE-encoding genome to a HE-lacking recipient. The *seg* and *mob* subtypes, which are also called freestanding endonucleases, belong to the GIY-YIG and HNH homing endonuclease families, respectively [16], a review]. The *seg*C, *seg*F, *seg*G, *mob*A, and *mob*E of T4 endonucleases are polycistronically transcribed with their respective upstream genes, whereas the endonuclease-specific promoters for *seg*A, *seg*G, *mob*C and *mob*D are immediately upstream of the endonuclease genes [16]. There is as yet no convincing evidence that the HEs can move across the boundary of species or genera. Nevertheless, these transposable genes may leave a trace of their involvement after the transfers. The sequence analysis for the Enterobacteria phage JSE intron revealed that the putative intron contained a truncated derivative of a HE gene [17], very similar to the truncated sequence in the intron of the T4 *nrd*B gene, suggesting that there is a rarely-detectable trace of the *mob*/*seg* elements in contemporary phage genomes [18].

We sequenced the genome of ϕpp2 – a new T4-like *Vibrio* phage with *mob* genes – which may be another paradigm in the plausible analysis of evolution of HE families in the bacteriophages and their hosts [8]. In the same host, *Vibrio parahaemolyticus*, the phage ϕpp2 can complement KVP40 in studying the genome spectra of the giant T4-related *Vibrio* phages.

## Methods

### Bacteria strains and growth conditions

*Vibrio* strains were bought from the Bioresource Collection and Research Center, Taiwan; including *V. alginolyticus* ATCC 17749, *V. carchariae* ATCC 35084, *V. damsela* ATCC 33536, *V. harveyi* ATCC 14126, *V. parahaemolyticus* ATCC 17802, *V. pelagius* ATCC 25916*,* and *V. vulnificus* BCRC15431. *V. parahaemolyticus* ATCC 17802 carries O1 serotype and no *tdh/trh* genes [19]. The *Vibrio* strains were maintained in Brain Heart Infusion (BHI) medium, supplemented with 3% NaCl. For long-term preservation, bacteria were frozen in BHI supplemented with 1% NaCl and 25% glycerol. When working, the strains were streaked onto the modified sea water yeast extract (rich MSWYE) agar plates consisting of 23.4 g NaCl, 6.98 g MgSO4^.^7H_2_O, and 0.75 g KCl in 1000 ml distilled water [19]. The pH was adjusted to 7.6 with 1 N NaOH, followed by addition of 5.0 g of proteose peptone (Difco), 3.0 g of yeast extract (Difco), and 20.0 g of agar per liter.

### Isolation and titer of bacteriophage

The water samples were collected from the aquaculture waterways around southern Taiwan. The enrichment procedure for the target phages has been described elsewhere [20]. In brief, 20% of MSWYE medium and 1% seed culture of *Vibrio parahaemolyticus* were added the micro-filtrated samples and incubated at 37°C for four hours to enrich the phages. In determining the phage concentrations, the bacterium *Vibrio parahaemolyticus* was freshly grown to 0.3–0.4 OD_600,_ in about two hours, and 200 μl of cells were added to 10 μl phages in a series of dilutions for infection, followed by the Agar Overlay Technique. The plaques were counted in 3–5 hours; the titers per ml were calculated by 100*(dilution factor)*(plaque counts).

### Electron microscopy

Preparation of phage particles for electron microscopy has been described elsewhere [20,21]. In brief, bacteriophage particles were applied onto parafilm to produce a spherical drop. Carbon-coated nitrocellulose films were fabricated on copper grids and placed face down on the sample drops for 1 min to absorb the particles. The samples were stained with freshly prepared 2% uranyl acetate (UA; Tris–HCl, pH 8.0) for 60 seconds. Images of phage particles were taken at a magnification of 40,000x, defocus of 3 μm, using a 200-kV electron microscope (JEOL JEM-2010, equipped with a Gatan-832 CCD camera).

### Analyses of bacteriophage DNA

In phage propagation, ten milliliters of ϕpp2 phage stock were added to 50 ml of *V. parahaemolyticus* (3x 10^8^ CFU ml^−1^) cultured in MSWYE, incubated in a shaker at 37°C for 3–5 hours, when the lysate was clear with some cell debris. The remaining cells and debris were removed by two centrifugations at 10000 × *g* for 30 minutes. With an optimal titer of 4 × 10^9^ PFU ml^−1^, the supernatant was stored at 4°C as a phage stock. To concentrate phages using a standard protocol with polyethylene glycol precipitation [22-24], solid NaCl (0.6 M) and polyethylene glycol 8000 (20%) were added and precipitation was performed overnight at 4°C. After centrifugation, the phage particles were resuspended in 2 ml of SM buffer and treated with DNase I and RNase A to remove contamination of host nucleotides. The polyethylene glycol was extracted by adding an equal volume of chloroform until the interface was clear. The aqueous phase containing phages was treated with Proteinase K and sodium dodecyl sulfate (SDS) at 56°C for 1 h. Phenol extraction was carried out three times at room temperature; the aqueous phase was further extracted with a 1:1 mixture of equilibrated phenol and chloroform. DNA precipitated by 2× volume of cold ethanol was re-dissolved in deionized water.

### Thermal stability of phage ϕpp2

Thermal stability tests have been described elsewhere [25,26]. Briefly, the bacterium *Vibrio parahaemolyticus* was freshly inoculated at the 1% volume of seed from overnight culture into 20 ml of rich MSWYE broth. When the cell density reached 0.4–0.5 OD_600_, the treated phages of a series dilution were added to infect the host for 5 minutes, mixed with top agar, and poured onto a solid surface of regular agar plate in order to count the plaques in 3−5 hours. 2 × 10^9^ PFU of phage particles were treated under 37−80°C and samples were taken at 15-min intervals. The supernatants from the centrifugation of 14000 × *g* for 3 minutes were diluted and titered for phage numbers by Agar-overlay method.

### Genome sequencing and annotation

Similar to shotgun sequencing described elsewhere, approximately 5 μg of the bacteriophage genomic DNA was randomly sheared by nebulization, and DNA sequencing was performed at Mission Biotech according to the manufacturer’s protocol for the Genome Sequencer GS Junior System (Roche Diagnostic). Low quality sequences of the reads generated by the GS Junior sequencer were trimmed off. *De novo* assembly of the shotgun reads was performed with the GS Assembler software. Sequence assembly and analyses were performed essentially as described previously [27]. Protein-coding genes (PEG) were predicted using The RAST Server (Rapid Annotations using Subsystems Technology*;*http://rast.nmpdr.org/) [28] and analyzed with the SEED- Viewer (http://www.theseed.org/wiki/Main_Page) [29]. Protein-coding genes were also checked using the *ab initio* gene-finding program Glimmer v3.02 [30]. rRNA genes of the draft assembly were identified using RNAmmer [31]. tRNA genes for all 20 amino acids that were predicted by the RAST were further verified using tRNAscan-SE [32]. Automatic functional annotation results obtained by the RAST were further compared with the proteins in the GenBank database using PSI-BLAST (http://www.ncbi.nlm.nih.gov/blast/Blast.cgi). The Neural Network Promoter Prediction (NNPP) program was used to find the promoters [33].

### Multiple sequence alignments

To determine the taxonomy status of the new phage isolate ϕpp2, the genome sequence data of Enterobacteria phage T4 and *Vibrio* phages KVP40 were employed to find the high homologous regions with the new phage after PSI-BLAST searches. Complete genome sequences of the *Vibrio* T4-like phages were acquired from NCBI, including Enterobacteria phage T4 (168903 bp in GenBank access no. NC_000866), *Vibrio* phage KVP40 (244834 bp in GenBank access no. NC_005083), *Aeromonas* phage 65 (235229 bp in GenBank access no. NC_015251), *Aeromonas* phage Aeh1 (233234 bp in GenBank access no. NC_005260), and *Prochlorococcus* phage P-SSM2 (252401 bp in GenBank access no. NC_006883). T4-like myoviruses also include Enterobacteria phages RB14 (NC_012638), RB16 (NC_014467), RB32 (NC_008515), RB51 (NC_012635), JS10 (NC_012741), and JSE (NC_012740), *Aeromonas* phages 325(NC_008208), and *Vibrio cholerae* phage ICP1 (NC_015157). PBCV-1 is the *Paramecium bursaria* Chlorella virus 1. Sequences of individual target genes retrieved from the genome sets were then aligned using ClustalW with default options [34]. The best alignments of individual genes were analyzed by a neighbor-joining method using the NEIGHBOR program in Phylogeny Inference Package (PHYLIP) [35]. Distances were calculated using the PROTDIST programs of PHYLIP and displayed in TreeView [36]. The ClustalW, PHYLIP, and TreeView were bundled in the BioEdit program version 7.0.5 [37].

## Results

### Phage morphology

**Figure 1:**
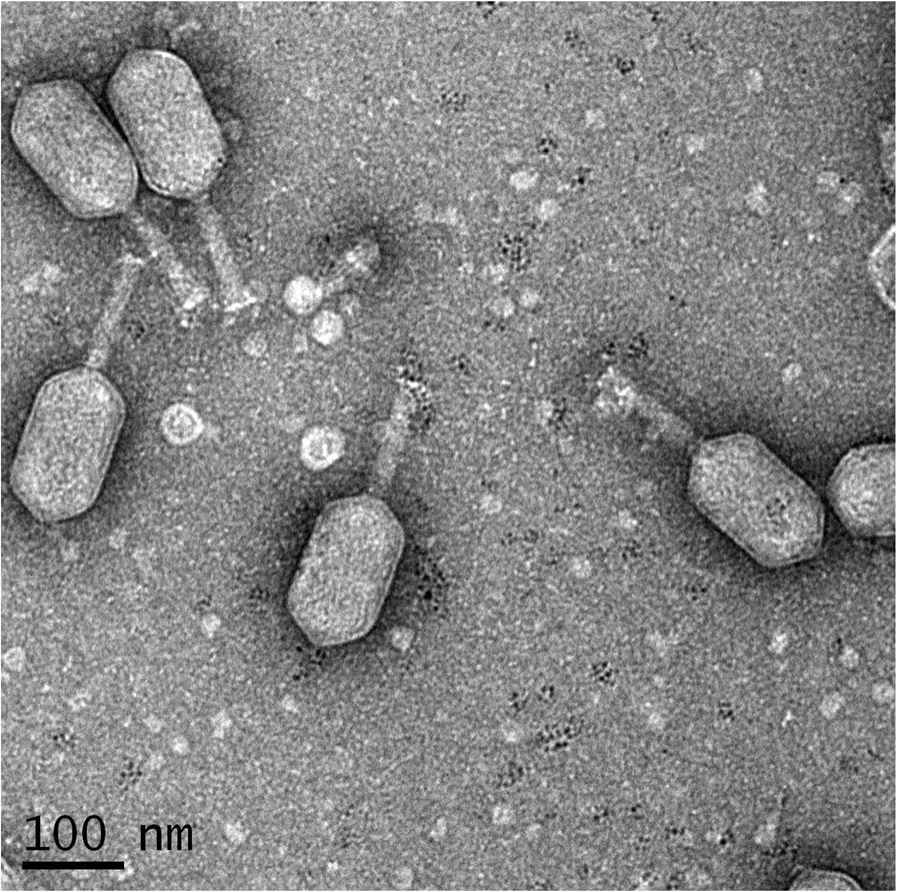
**Transmission electron micrograph of phage ϕpp2particles with several structural proteins**. The phage particles were purified with three times of centrifugations by PEG-NaCl precipitation method mentioned in the Material section. Virion particles were negatively stained with uranyl acetate for EM. The bars represent a length of 100 nm.

The morphology of phage ϕpp2 was observed by transmission electron microscopy, which is traditionally one of the most frequently used methods to classify phages. As Figure 1 shows, ϕpp2 was a large phage with nonenveloped head, neck, collar, and tail: the head was approximately 90–95 nm wide by 150–160 nm long and the tail was about 110–120 nm long with 20–25 nm in diameter. A baseplate and tail pins were observed under different focus, while long tail-fibers were threading randomly.

### Host range

The susceptibility of seven *Vibrio* strains to the phage ϕpp2 was also investigated with the Agar-overlay method. Among them, *V. parahaemolyticus*, *V. damsela* and *V. alginolyticus* were found susceptible to phage ϕpp2 while the other four species (*V. carchariae*, *V. damsela*, *V. harveyi*, *V. pelagius,* and *V. vulnificus*) could not be infected even at high MOI.

### Viability of phage ϕpp2 in the thermal environment

**Figure 2:**
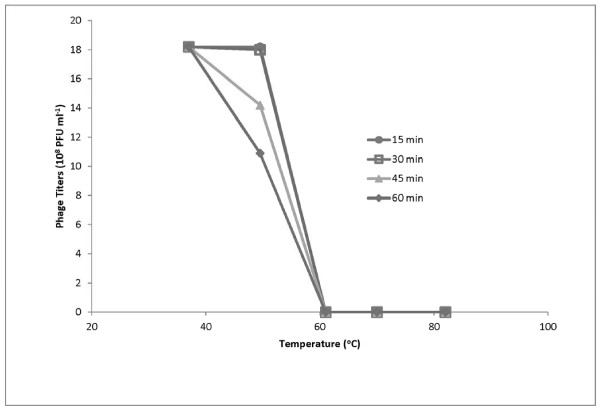
Thermal stability tests of the phage ϕpp2. Samples were taken at different time intervals to titer the phage particles of infectivity.

Thermal stability test was carried out to analyze the heat-resistant capability of phage ϕpp2 at pH7.5–8.0. The phage was incubated at 37, 50, 61, 70, and 80°C for one hour, respectively. As Figure 2 shows, the phage titers at different time intervals demonstrated that phage ϕpp2 stock solution retained almost 100% infection activity after incubation at temperatures lower than 37°C for one hour. When the temperatures rose above 50°C, viability of phage ϕpp2 declined; about 60% phages remained alive after being heated for 60 minutes. At temperatures over 60°C, nearly all phages were inactivated after 15 minutes of incubation.

### Genome organization and annotation

The genome sequence of *Vibrio* phage ϕpp2 was determined using the Roche Genome Sequencer system (454 Life Sciences, Branford, CT). A total of 21,452 reads and 7,985,781 bases, with an average length of 372.3 bases, were obtained. After *de novo* assembly among at least 40 nucleotide overlap with minimum overlap identity of 90%, the whole genome was aligned to one single contig, with coverage of 32-fold and the Q_40_ Plus Bases of 98.89% (where Q_40_ represents an error rate of 99.99%). Currently, the draft genome has a total of 246,421 bp, which includes 270 nt of Q_39_ Minus Bases (0.11%). The GenBank access number for this new genome is assigned to be JN849462.

The genome size of the *Vibrio* phage ϕpp2 is 1587 bp larger than 244,834 bp of KVP40 bp and far bigger than the 168,903 bp of T4, while its average G + C content was 42.55%, which is the same as the 42.60% of KVP40 but not as the 35.3% of T4. No rRNA genes of the draft assembly were identified using RNAmmer. Sixty tRNA overlap genes that were preliminarily predicted by the RAST were further verified to be 30 using tRNAscan-SE. In annotation for protein coding regions, 30 subsystem features were predicted by the SEED-RAST server, including 15 features which were relevant to phage structure proteins, 2 for phage DNA synthesis, 7 for nucleotide reactions, and one each for fluoroquinolone resistance, protein degradation and RNA metabolism. One possible gene for resistance of beta-lactamase was not included by the auto-annotation.

### Large indels (insertion/deletions)

Overall of *Vibrio* phage ϕpp2 was similar to the genome organization of vibriophage KVP40 and Enterobacteria phage T4 (Table 1). In comparison with KVP40, 15 deletions and 19 insertions were found in ϕpp2, of which 25 indels only affected one single ORF. It is noteworthy that a single deletion occurred in the *seg*D-type HE (PEG145 of KVP40), at the junction of KVP40.0145 (at 84923.85078) and KVP40.0146 (complement 85073.85768), implying that ϕpp2 had lost this HE. Most of the indel sizes were in the range around 100–400 nt; nevertheless, some large replacements existed, i.e., 621 nt at KVP40.0102 (61372.61992), 702 nt at KVP40.0121 (70639.71307), 687 nt at KVP40.0147 (85926.86240), 664 nt at KVP40.0172 (98546.98713), 672 nt at KVP40.0277 (*nrd*A, 146553.148778), and 693 nt at KVP40.0315 (178766.178930). Additionally, three KVP40 genes were replaced by giant inserts in ϕpp2: 1098 nt of ϕpp2 replaced the gene near KVP40.0363 (gp23, 224506.226050, 1545 nt), 1143 nt of ϕpp2 replaced the gene at KVP40.0263 (137878.138114, 237 nt), and 3330 nt of ϕpp2 replaced the gene at KVP40.0297 (complement 160413.160988,576 nt). The three giant indels signified that the *Vibrio* phage ϕpp2 was a new species from KVP40.

**Table 1:** **Gene functions of the *Vibrio *phage ϕpp2**


Feature ID	Start	Stop	nt (bp)	aa	Function	Match to	Color
fig 75320.3.peg.1	41	973	933	311	RNaseH ribonuclease	KVP40 & T4	G*
fig 75320.3.peg.3	1318	1611	294	98	late promoter transcription accessory protein	KVP40	Y
fig 75320.3.peg.6	3216	3761	546	182	Frd dihydrofolate reductase	KVP40 & T4	G
fig 75320.3.peg.7	3758	4477	720	240	ATP-dependent Clp protease proteolytic subunit (EC3.4.21.92)	KVP40	Y
fig 75320.3.peg.8	4544	5644	1101	367	Phage recombination protein	KVP40 & T4	G
fig 75320.3.peg.10	6060	7343	1284	428	DNA primase-helicase subunit	KVP40 & T4	G
fig 75320.3.peg.12	7583	9418	1836	612	Ribonucleotide reductase of class III (anaerobic), large subunit (EC 1.17.4.2)	KVP40 & T4	G
fig 75320.3.peg.15	10506	10982	477	159	Ribonucleotide reductase of class III (anaerobic), activating protein (EC 1.97.1.4)	KVP40 & T4	G
fig 75320.3.peg.16	10982	11506	525	175	putative serine/threonine protein phosphatase	KVP40	Y
fig 75320.3.peg.18	12420	13244	825	275	98.1% KVP40 DNA helicase, phage-associated	KVP40	Y
fig 75320.3.peg.19	13244	13720	477	159	gp61.1 conserved hypothetical	KVP40 & T4	G
fig 75320.3.peg.20	13801	14859	1059	353	DNA primase (EC 2.7.7.-)/DNA helicase (EC 3.6.1.-). Phage-associated	KVP40 & T4	G
fig 75320.3.peg.21	14859	15356	498	166	Deoxyuridine 5'-triphosphate nucleotidohydrolase (EC 3.6.1.23)	KVP40	Y
fig 75320.3.peg.23	15589	16281	693	231	exonuclease A	KVP40 & T4	G
fig 75320.3.peg.31	18182	19081	900	300	Thymidylate synthase (EC 2.1.1.45)	KVP40	G
fig 75320.3.peg.42	23359	24024	666	222	NAD-dependent protein deacetylase of SIR2 family	KVP40	Y
fig 75320.3.peg.43	24180	25973	1794	598	DNA gyrase subunit B (EC 5.99.13)	KVP40 & T4	G
fig 75320.3.peg.49	27350	28078	729	243	Ser/Tar protein phosphatase family protein	KVP40	Y
fig 75320.3.peg.55	30282	31625	1344	448	DNA ligase	KVP40 & T4	G
fig 75320.3.peg.60	36003	36242	240	80	glutaredoxin	KVP40	Y
fig 75320.3.peg.61	36301	37197	897	299	Phage capsid vertex protein (T4-like gp24)	KVP40 & T4	G
fig 75320.3.peg.62	37206	37718	513	171	T4-like phage RNA polymerase sigma factor for late transcription # T4-like phage gp55#T4GC0140	KVP40 & T4	G
fig 75320.3.peg.68	41040	41504	465	155	gp30.3	KVP40 & T4	G
fig 75320.3.peg.69	41509	41994	486	162	Putative 5'(3')-deoxyribonucleotidase (EC 3.1.3.-)	KVP40	Y
fig 75320.3.peg.70	41991	43031	1041	347	Phage recombination-related endonuclease Gp47	KVP40 & T4	G
fig 75320.3.peg.72	43248	45485	2238	746	recombination endonuclease subunit	KVP40 & T4	G
**fig 75320.3.peg.75**	**46247**	**46912**	**666**	**222**	**27.11% T4 Sliding clamp DNA polymerase accessory protein, phage associated # Gp45**	**T4**	**DG**
fig 75320.3.peg.76	46983	47939	957	319	Replication factor C small subunit/Phage DNA polymerase clamp loader subunit # T4-like phage gp44 # T4 GC0157	KVP40 & T4	G
**fig 75320.3.peg.77**	**47950**	**48438**	**489**	**163**	**31.11% T4 Phage DNA polymerase clamp loader (fig|10665.1.peg.49)**	**T4**	**DG**
fig 75320.3.peg.78	48473	48853	381	127	RegA translaticnal repressor of early genes	KVP40 & T4	G
**fig 75320.3.peg.79**	**49482**	**48856**	**627**	**209**	**MobE homing endonuclease**	**T4**	**R**
fig 75320.3.peg.81	50130	52682	2553	851	DNA polymerase	KVP40 & T4	G
fig 75320.3.peg.83	53047	54192	1146	382	Rn1A	KVP40 & T4	G
fig 75320.3.peg.88	55585	56742	1158	386	3'-phosphatase, 5'-polynucleotide kinase, phage-associated #T4-like phage Pset #T4 GC1648	KVP40 & T4	G
fig 75320.3.peg.116	69766	70218	453	151	CMP/dCMP deaminase, zinc-binding	KVP40 & T4	G
fig 75320.3.peg.117	70273	71199	927	309	NADPH-dependent 7-cyano-7-deazaguanine reductase (EC 1.7.1.-)	KVP40	Y
fig 75320.3.peg.118	71268	71942	675	225	GTP cyclohydrolase I (EC 3.5.4.16) type 1	KVP40	Y
**fig 75320.3.peg.119**	**72615**	**71914**	**702**	**234**	**Phage-associated homing endonuclease**	**T4**	**R**
fig 75320.3.peg.121	74083	74991	909	303	NADPH-dependent 7-cyano-7-deazaguanine reductase (EC 1.7.1.-)	KVP40	Y
fig 75320.3.peg.122	75047	75763	717	239	Queuosine Biosynthesis QueC ATPase	KVP40	Y
**fig 75320.3.peg.124**	**76380**	**76655**	**276**	**92**	**32.1% T4 Phage tail fibers**	**T4**	**DG**
fig 75320.3.peg.127	77160	77498	339	113	Phage capsid and scaffold	KVP40	Y
fig 75320.3.peg.129	78455	77946	510	170	gp49 recombination endonuclease VII	KVP40 & T4	G
fig 75320.3.peg.131	78799	79806	1008	336	RNA ligase, phage-associated	KVP40 & T4	G
fig 75320.3.peg.141	83194	85263	2070	690	Phage rIIA lysis inhibitor	KVP40 & T4	G
fig 75320.3.peg.142	85256	86293	1038	346	rIIB protector from prophage-induced early lysis	KVP40 & T4	G
fig 75320.3.peg.148	89179	90216	1041	347	NrdC 1 1 conserved hypothetical protein.	KVP40 & T4	G
fig 75320.3.peg.151	90946	92211	1266	422	Dda DNA helicase	KVP40 & T4	G
fig 75320.3.peg.157	94752	95777	1026	342	Nicotinamide-nucleotide adenylyltransferase, NadM family (EC 2.7.7.1)/ADP-ribose pyrophosphatase	KVP40	Y
fig 75320.3.peg.188	105782	106366	585	195	Thymidine kinase (EC 2.7.1.21)	KVP40 & T4	G
fig 75320.3.peg.201	111295	111711	417	139	endonuclease	KVP40 & T4	G
fig 75320.3.peg.208	113557	114537	981	327	Nicotinamide-nucleotide adenylyltransferase, NadR family (EC 2.7.7.1)/Ribosyln:cotinamide kinase (EC 2.7.1.22)	KVP40	Y
fig 75320.3.peg.212	115492	116157	666	222	Ribosyl nicotinamide transporter, PnuC-like	KVP40	Y
fig 75320.3.peg.249	130608	131138	531	177	Cell wall mannoprotein with similarity to Tir1p, Tir2p, Tir3p, and Tir4p; expressed under anaerobic conditions, comp	KVP40	Y
fig 75320.3.peg.255	134377	135366	990	330	moa A/nifB/pqqE family protein	KVP40	Y
fig 75320.3.peg.256	136483	135359	1125	375	moa A/nifB/pqqE family protein	KVP40	Y
fig 75320.3.peg.260	138690	140183	1494	498	Nicotinamide phosphoritosyltransferase (EC 2.4.2.12)	KVP40	Y
fig 75320.3.peg.268	144971	143919	1053	351	moaA/nifB/pqqE family protein	KVP40	Y
fig 75320.3.peg.273	147028	149253	2226	742	Ribonucleotide reductase of class Ia (aerobic), alpha subunit (EC 1.17.4.1)	KVP40 & T4	G
***fig 75320.3.peg.274***	***149293***	***149964***	***672***	***224***	***Phage-associated homing endonuclease***	***T4***	***R***
fig 75320.3.peg.275	149957	151081	1125	375	Ribonucleotide reductase of class Ia (aerobic), beta subunit (EC 1.17.4.1)	KVP40 & T4	G
fig 75320.3.peg.276	151083	151382	300	100	NrdC thioredoxin	KVP40 & T4	G
fig 75320.3.peg.279	152313	153317	1005	335	Thioredoxin, phage-associated	KVP40 & T4	G
fig 75320.3.peg.280	153363	154649	1287	429	gp52 topoisomerase II medium subunit	KVP40 & T4	G
fig 75320.3.peg.282	154900	155781	882	294	Queuosine Biosynthesis QueE Radical SAM	KVP40	Y
fig 75320.3.peg.293	160936	161235	300	100	anti-sigma70 protein	KVP40	Y
fig 75320.3.peg.296	165535	162206	3330	1110	Phage tail fibers (Match to KVP40 peg.297)	KVP40	Y
fig 75320.3.peg.297	168882	165607	3276	1092	tail fiber fragment	KVP40	Y
fig 75320.3.peg.322	184667	184212	456	152	gp57B conserved hypothetical protein	KVP40 & T4	G
fig 75320.3.peg.324	185581	184943	639	213	dNMP kinase	KVP40 & T4	G
fig 75320.3.peg.325	186347	185814	534	178	gp3 tail completion and health stabilizer protein	KVP40 & T4	G
fig 75320.3.peg.328	189332	188484	849	283	Phage baseplate hub	KVP40 & T4	G
fig 75320.3.peg.329	190090	189344	747	249	Phage baseplate-tail tube initiator	KVP40	Y
**fig 75320.3.peg.330**	**190507**	**190094**	**414**	**138**	**55.97% T4 Phage DNA end protector during packaging**	**T4**	**DG**
fig 75320.3.peg.331	191144	190689	456	152	Phage head completion protein	KVP40 & T4	G
fig 75320.3.peg.332	191211	192350	1140	380	Phage baseplate tail tube cap	KVP40 & T4	G
fig 75320.3.peg.333	192350	192928	579	193	Phage baseplate wedge	KVP40 & T4	G
fig 75320.3.peg.335	194207	195466	1260	420	Phage baseplate hub	KVP40 & T4	G
fig 75320.3.peg.337	195956	196252	297	99	PAAR	KVP40 & T4	G
fig 75320.3.peg.340	197717	198136	420	140	Phage baseplate wedge	KVP40 & T4	G
fig 75320.3.peg.341	198223	200181	1959	653	Phage baseplate wedge	KVP40 & T4	G
fig 75320.3.peg.342	200181	203678	3498	1166	Phage baseplate wedge initiator	KVP40 & T4	G
fig 75320.3.peg.343	203680	204702	1023	341	Phage baseplate wedge	KVP40 & T4	G
fig 75320.3.peg.344	204758	205714	957	319	gp9	KVP40 & T4	G
fig 75320.3.peg.345	205724	207970	2247	749	Phage baseplate wedge	KVP40 & T4	G
fig 75320.3.peg.348	210191	211612	1422	474	prophage LambdaSa04, minor structural protein, putative	KVP40 & T4	G
fig 75320.3.peg.349	211911	213590	1680	560	Phage neck whiskers	KVP40 & T4	G
fig 75320.3.peg.350	213601	214524	924	308	Phage neck protein	KVP40 & T4	G
fig 75320.3.peg.351	214528	215364	837	279	Phage neck protein	KVP40 & T4	G
fig 75320.3.peg.352	215593	216726	1134	378	tail health stabilizer and completion protein	KVP40 & T4	G
fig 75320.3.peg.354	217441	217989	549	183	Phage terminase, small subunit	KVP40 & T4	G
fig 75320.3.peg.355	217949	219751	1803	601	Phage terminase, large subunit	KVP40 & T4	G
fig 75320.3.peg.356	219798	221813	2016	672	Phage tail health monomer	KVP40 & T4	G
fig 75320.3.peg.357	221864	222364	501	167	Phage tail fibers	KVP40 & T4	G
fig 75320.3.peg.358	222404	223951	1548	516	portal vertex protein of head	KVP40 & T4	G
fig 75320.3.peg.360	224131	224622	492	164	Phage capsid and scaffold	KVP40 & T4	G
fig 75320.3.peg.361	224625	225266	642	214	Phage prohead core scaffold protein and protease	KVP40 & T4	G
fig 75320.3.peg.362	225299	226141	843	281	Phage scaffold prohead core protein	KVP40 & T4	G
fig 75320.3.peg.363	226212	227756	1545	515	Phage major capsid protein	KVP40 & T4	G
fig 75320.3.peg.364	228910	227813	1098	366	PSI-BLAST tRNA nucleotidyltransferase (Acinetobacter baumannii & Pseudomonas fluorescens)	KVP40 & T4	G
fig 75320.3.peg.367	230824	231315	492	164	Inh	KVP40 & T4	G
fig 75320.3.peg.377	236908	238431	1524	508	DNA helicase, phage-associated	KVP40 & T4	G
fig 75320.3.peg.381	239752	239339	414	138	UvsY recombination, repair and ssDNA binding protein	KVP40	Y
fig 75320.3.peg.383	246382	242612	3771	1257	gp34 long tail fiber, proximal subunit	KVP40	Y

### Gene functions

**Figure 3:**
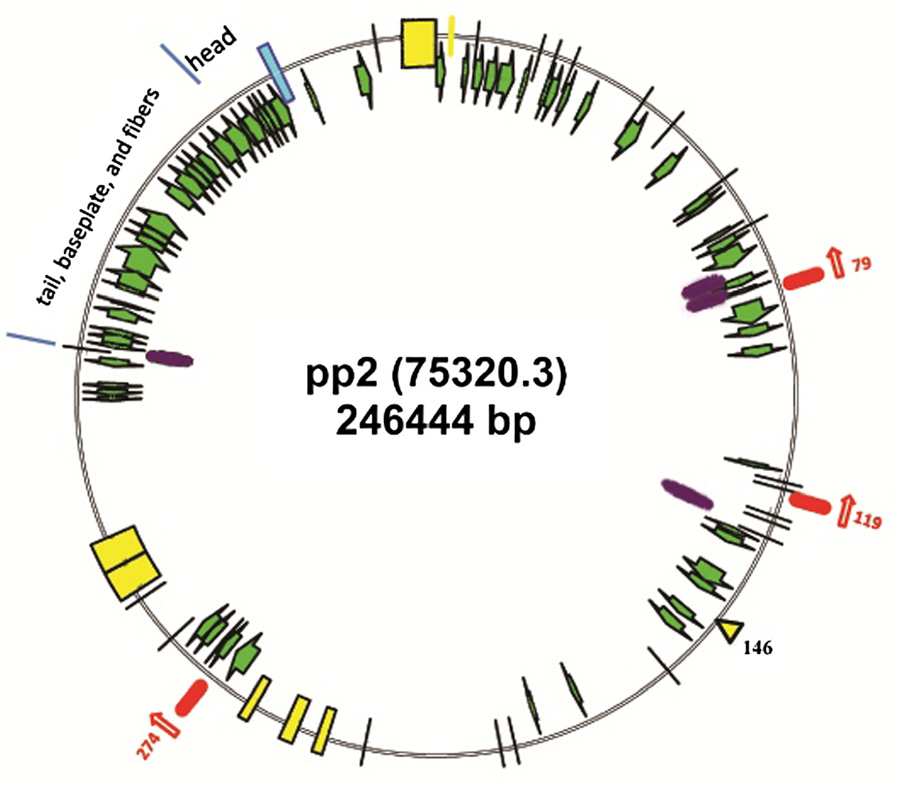
**Genome map of the *Vibrio *phage ϕpp2.** Green arrows indicate the genes matched to both Enterobacteria phage T4 and Vibrio phage KVP40. Yellow ticks on the circle indicate that the genes fitted to KVP40 only while the yellow triangle indicates the absent site for KVP40.0146 HE gene. Purple represents that the genes only aligned well with T4. The cyan is for one gene matched to GTP cyclohydrolase I from *Bdellovibrio bacteriovorus* HD100, *Vibrio angustum* S14, and *Cytophaga hutchinsonii* ATCC 33406. Red bars with the number indicate the PEG numbers of potential HE.

With the extracting plausible protein sequences encoded by the genomic DNAs, 383 PEGs were found in *Vibrio* phage ϕpp2, in contrast to 381 PEGs for KVP40 found with the same RAST method (Table 1). Functions were identified by sequence similarity (Table 1). 104 (27.2%) out of 383 PEGs were matched to known functions of T4-like phage genes and assorted bacteria genomes, while functions of 279 (72.8%) PEGs were still unknown. Among these, as Figure 3 and Table 1 show, 67 PEGs were matched to both T4 and KVP40 (green arrows), 29 PEGs to KVP40 alone (yellow ticks), 7 PEGs to other T4-like (purple and red ticks), and one to assorted bacteria (cyan). Between the genomes of ϕpp2 and KVP40, the similarity of 254 genes was greater than 94%, whereas 17 PEGs of KVP40 and 21 PEGs of ϕpp2 were unmatched to any known, in addition to 15 genes with lower similarity ( Additional file 1). At least 29 PEGs (7.6%) were directly related to phage particle structures, such as head, tail, and baseplate. ϕpp2 uniquely possessed the proteins involved in potassium channel, gp2 (DNA end protector), tRNA nucleotidyltransferase, and mob-type HEs, which were not reported in the case of KVP40. Several genes were split: in ϕpp2, PEG297 shared paralogs with PEG296, as the same pattern for PEG119 sharing with PEG274, while KVP40.0089 (54956.55189) and KVP40.0090 (55200.56117) paralogs were matched to one single ϕpp2 PEG88.

### Transfer RNAs

The RAST predicted 60 pieces of potential tRNAs, spanning in the range of 9175 bp in *Vibrio* phage ϕpp2, while in KVP40 29 tRNAs were found in the range of 8702 bp. Using tRNAscanSE to recalculate the structures with overlapping sequences, 30 tRNAs in the cluster were double verified for *Vibrio* phage ϕpp2 while 29 tRNAs remained for KVP40; both contained three pseudo-forms of low score for GCA (two) and TGC (one) anticodons. The *Vibrio* phage ϕpp2 tRNA cluster encoded for 17 amino acid codons, but there were no anticodons for alanine, glutamine, and tyrosine. The KVP40 tRNA region was 475 bp shorter than the ϕpp2 but shared 97% similarity over the cluster. A big insert of 465 nt in the middle of the cluster created no putative tRNA structure in the range of insert. In the *Vibrio* phage ϕpp2, one extra met-tRNA, which formed from the 28 nt mutation out of 72 nt, was created at the upstream of junction that was 6 nt upstream from the 465-nt insert.

### Searching *mob*-like genes and neighbors

**Figure 4:**
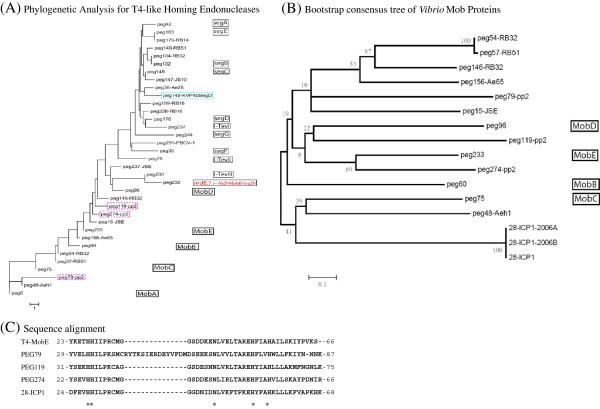
**Phylogenetic analyses and similarity of the HE genes from different T4-related phages**. ϕpp2 is a *Vibrio* phage isolated in this study. PEG numbers without dash are Enterobacteria phage T4. The homing endonucleases are named with gene product numbers followed by the dash lines for the hosts of the phages: Enterobacteria phages include RB14, RB16, RB32, RB51, JS10, and JSE; *Aeromonas* phages, Aeh1, 25 and 65; PBCV-1 is *Paramecium bursaria* Chlorella virus 1; and *Vibrio* phage ICP1. (**A**) Rooted phylogenetic tree for the homing endonucleases of *Vibrio* and T4-like phages by PROTDIST-neighbor joining method; the amino acid sequences were aligned with BLOSUM62 matrix, gap penalty = 8 and extension penalty = 2. (**B**) Bootstrap analysis for the Mob-type HEs of the *Vibrio* phages against T4 phages. The bootstrap values of percentages in 1000 replicates are placed on the branch for the nodes defining each monophyletic clade. The scale bars represent distance length. (**C**) H-N-H alignment of three HE genes from ϕpp2 with T4 mobE and ICP1 ORF28 (a phage in *Vibrio cholerae*).

In sequence similarity analysis by PSI-BLAST, three paralog genes of homing endonucleases were found in the *Vibrio* phage ϕpp2: PEG79, PEG119, and PEG274, in which the number of amino acid residues was 209, 234, and 224 aa, respectively. The PEG119 and PEG274 were aligned to neighborhood of T4 MobE and close to MobD (Figure 4A). The PEG79 were situated next to the group of MobA (Figure 4A). The PEG119 shared 37% similarity with PEG274, while PEG79 shared 27% and 35% with the other two in pair-wide alignment of amino acid sequences. In Bootstrap analysis with 1000 replicates, the branch percentage showed that the three PEGs in ϕpp2 were all Mob-like homing endonucleases, least likely to be a GIY-YIG type (Figure 4B). Although low overall similarity was found between them, all three PEGs aligned the H-N-H motif very well in their N-termini (Figure 4C). First two His-32 and His-33 in PEG79 were highly conserved within the motif of ExHHILPK for PEG119 and PEG274. The second Asn-50 of PEG79 was situated in the motif of SDExNLV, and the third His was paired as HxxxH found in the motif of LTAREH---HxLLxK.

We identified the Mob types of ϕpp2 around the genome according to the orientation similarity to the neighbor ORFs of 15 homing endonucleases in Enterobacteria phage T4: gt (glucosyl transferase) and *nrd* (ribonucleotide reductase) orthologs. The details of the search methods are described in Additional file 2. No match to T4 α.gt or β.gt was found in entire genomes of ϕpp2 and KVP40 (NC_005083); therefore, the *mob*B-like gene could not exist in ϕpp2. Four *nrd*-like genes were found in ϕpp2: one was found explicitly by the RAST and three others were implicit but manually confirmed with PSI-BLAST searches. The PEG12 protein was similar to the large subunit of anaerobic ribonucleotide reductase of class III (EC 1.17.4.2), with 52.05% similarity to T4 *nrd*G, while PEG15 was assumed to be the activating protein (EC 1.97.1.4) for the ribonucleotide reductase with 52.74% similarity to T4 *nrd*D. PEG132, which was matched to T4p232 in the boundary of MobE and downstream close-by *seg*D, was denoted as *nrd*B.1. The fourth *nrd*-like gene, 1041 nt of PEG148 (347 aa, 89176.90216) in ϕpp2, was mapped to T4 nrdC.11.

Using the neighbor-indirect method (details in Additional file 2), the neighbors of T4 *mob* genes were mapped back to ϕpp2 genome. The neighbor gene T4p074 (*nrd*G) of *mob*C (T4p075) was back-projected to ϕpp2 PEG15 with a similarity of 52.05%; another neighbor gene T4p076 (*nrd*D) was matched to ϕpp2 PEG12 with a similarity of 52.74%. The distance of the PEG12/15 pair was at least 37874 nt apart from the PEG79 – it was even farther to PEG 119 and PEG274. Similarly, the PEG132 and PEG148 were still too far to be adjunction neighbors for all three potential ϕpp2 *mob* genes, i.e., PEG132 was 7895 nt apart from PEG119.

**Figure 5:**
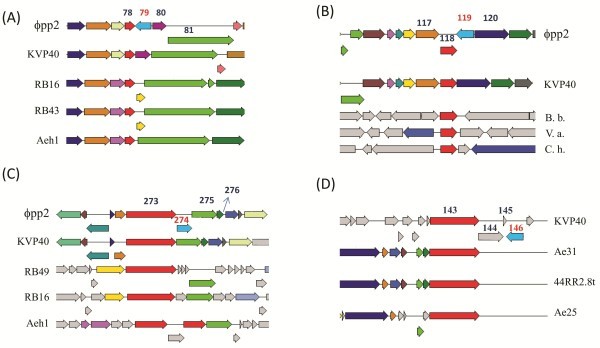
**The best aligned T4-related phage genes for the neighbor genes of ϕpp2 and KVP40 HEs using the neighbor-direct method**. The approach is described in the text and Additional file 2. The same color arrows represents the homologous genes. The cyan arrows indicate the HE genes for ϕpp2 and KVP40. (**A**) Neighbors of ϕpp2 PEG79: 78 for RegA translational repressor of early genes, 80 for phage hypothetical protein, and 81 for DNA polymerase. (**B**) Neighbors of ϕpp2 PEG119: 118 for GTP cyclohydrolase I from *Bdellovibrio bacteriovorus* HD100, *Vibrio angustum* S14, and *Cytophaga hutchinsonii* ATCC 33406; 120 for phage hypothetical protein. (**C**) Neighbors of ϕpp2 PEG274: 273 & 275 for Nrd, ribonucleotide reductase Ia; 276 for NrdC thioredoxin. (**D**) Neighbors of KVP40.146: 143 for rIIA protector; 144 for rIIB protector.

Alternatively, using the so-called neighbor-direct method ( Additional file 2), the *mob*-neighbor genes of ϕpp2 PEG79, PEG119, and PEG274 were manually *de novo* searched with PSI-BLAST. Neither neighbors of PEG79 (upstream PEGs 70 ~ 78 and downstream PEGs 80 ~ 90) nor of PEG119 (upstream PEGs 110 ~ 117 and downstream PEGs 120 ~ 125) were in any way close to *nrd*-like genes (Figure 5A and B).

### *Vibrio* phage ϕpp2 PEG274 with *mob*E-type neighbors

In *de novo* identification of a *mob*-type for ϕpp2 PEG274 (672 nt) using the neighbor-direct method, ϕpp2 PEG273 (2226 nt) of the upstream neighbor gene was blasted to NrdA of *Aeromonas* phages (PX29 and phiAS5), Enterobacteria phages (JSE, RB49, phi1, and T4), and *Shigella* phage SP18 (Figure 5C). The downstream neighbor PEG275 (1125 nt) was blasted to NrdB of *Aeromonas* phages phiAS5 and Aeh1, *Klebsiella* phage KP15, and Enterobacteria phage RB16. Another neighbor, PEG276 (300 nt), was also blasted to the NrdC thioredoxin; it aligned well as 86% homologous to NrdC thioredoxin in *Aeromonas* phages phiAS5, Aeh1, and 65, as well as to *Klebsiella* phage KP15, *Shigella* phage SP18, and Enterobacteria phages RB16, RB43, and ime09. With the matches of upstream and downstream of *nrd*-like genes which complemented the full organization of MobE neighbors, the ϕpp2 PEG274 can be annotated as a MobE-type HE, without the existence of *I-Tev*III intron.

### Expression of ϕpp2 PEG 274 gene

All homing endonucleases of ϕpp2 and KVP40 started with an AUG initiation codon. For ϕpp2 PEG 274, AGGA as a ribosome binding site (RBS) was optimally situated 6 nt upstream of the PEG start codon while translation initiation regions are not positioned at the optimal distance of 6–9 nucleotides from the AUG codon for PEG79 and PEG 119. The AAGAGAG for PEG79 was not a good match to antisense of small rRNA while the predicted PEG119 RBS AGGA is immediately adjunct to the AUG codon, which is rarely considered to be a good initiation site for translation.

As shown in Figure 3, the direction of transcription for PEG79 and PEG119 was counter-clockwise, whereas the PEG274 promoter was clockwise, which was the same as most T4 homolog genes. The NNPP predicted several promoters of high scores (>0.95) in the upstream of these three genes. It is worth noting that three promoters were identified around PEG274, the aforementioned MobE-type homing endonuclease. In contrast to the translational initiation AUG position of PEG274 at 149293–149964 in the genome of ϕpp2, the nearby promoters were also positioned at 148783 (510 nt upstream; pR148783), 149272 (21 nt immediately upstream; pR149272), and 149974 (10 nt downstream; pR149974). pR149272 was the best fit to the promoter consensus, which consisted of TTGTGA for −35 box and ATGTAAAAT for −10 box. Accompanying this promoter, some weak binding sites for transcription factors were also observed: TGTAAAAT for *rpo*D17 at position 149258, ATATAAAT for *arg*R2at 149264, and GTTCATAT for *tor*R at 149273.

## Discussion

Electron microscopy revealed that the phage ϕpp2 particles were morphologically similar to T4 phage and vibriophage KVP40, which is a long head (~140 nm long and ~70 nm wide) with a prolate icosahedral capsid and a contractile tail with associated baseplate and extended tail fibers. ϕpp2 is most likely type A phage in Bradley’s classification of *Myoviridae*[38], based on the morphological characteristics (Figure 1). The protein profiles in ϕpp2 contain a heavy band of ~50 kD, which is similar to known T4 structure proteins of major capsid protein (data not shown). With hourly heat-tolerance at 50°C (Figure 2), this phage could infect aquaculture pathogens, *V. parahaemolyticus*, *V. damsela* and *V. alginolyticus.* The complete genome of the new *Vibrio* phage ϕpp2 was sequenced (GenBank access_no JN849462), which was a sibling phage of KVP40 but with different HE genes (Figure 3).

In the phylogenetic tree (Figure 4A), the PEG79 was distantly situated next to the group of MobA. Although their overall similarity was low, the N-termini of all three PEGs aligned well with the H-N-H motif (Figure 4C). The first His-32/33 in PEG79 was highly conserved within the motif of ExHHILPK for PEG119 and PEG274. The second Asn was situated in the motif of SDExNLV and the third His-pair was in the paired form of HxxxH found in the motif of LTAREH---HxLLxK. This reveals that the ϕpp2 HE genes belong to Mob-type because the H-N-H is the critical motif for the enzyme activity [10]. The vibriophage KVP40 carries segD/C (KVP40.0146) [8]. *V. cholerae* ORF80 in ICP1 belongs to segG (data not shown) while another ICP1-ORF28 is closely related to MobC (Figure 5B and C) [9].

By PSI-BLAST directly from the neighbor genes of ϕpp2 PEG274 (the neighbor-direct method), PEG273, PEG275, and PEG276 were highly homologous to NrdA, NrdB and NrdC thioredoxin in *Aeromonas* phages, Enterobacteria phages, *Klebsiella* phage KP15 and *Shigella* phage SP18, respectively. With match of both up- and downstream, together with the conserved motif of HE in Figure 4C, the PEG274 can be annotated as MobE-type HE. For PEG274 protein expression, we found a good promoter (pR149272) immediately upstream of the PEG274 gene; thus, the promoter was considered as endonuclease-specific. The transcript of PEG274 mRNA was also equipped with a good consensus of ribosome binding site AGGA at 6 nt upstream of the start codon AUG.

Sequence of ϕpp2 PEG79 was comparatively similar to MobA gene, but PEG79 was flanked by DNA*pol* and *reg*A (phage endoribonuclease translational repressor of early genes; Figure 5A), where they do not neighbor any *mob* genes in T4. The PEG119 and PEG79 genes were similar to T4p232 and T4p233 (*mob*E), respectively. The landmark of T4p131 (e.8, complement 70360.70623) is also very similar to PEG275. In other words, three ϕpp2 *mob*-like genes (PEG79, PEG119, and PEG274) would be mapped onto the cluster of *I-Tev*III-*nrd*B1-*mob*E located at T4p130 to T4p133 in T4 genome [16], a review]. This implies the characteristics of HE mobility.

KVP40.0146 (696 nt) encoding 231 aa was PSI-BLAST to GIY-YIG endonuclease genes, including *Aeromonas* phages (phage 25 and phiAS5), *Acinetobacter* phages (Acj61 and Ac42), Chlorella virus FR483, Enterobacteria phages (RB51, RB16, T4), *Klebsiella* phage KP15, and *Staphylococcus* phage PH15. As shown in Figure 4A, the phylogenetic analysis plotted KVP40.0146 to be a *seg*C/D type. Using the neighbor-direct method ( Additional file 2), KVP40.0144 and KVP40.0145 could not be matched to any protein of known function (Figure 5D) while KVP40.142 and KVP40.143 could be similar to rIIA/B lysis protectors. Both were too distant to bracket the KVP40.0146 of GIY-YIG endonuclease gene for mimicking the T4 segD neighbor. In T4 HEs, types of *mob*C, *mob*D, and *mob*E can be classified by neighbor elements as well as different arrangements of their promoters: nrdD-mobC-nrdG, mobD-nrdC.11, and nrdA-(I-*Tev*III)-mobE-nrdB, respectively [16]. In KVP40, there are seven *nrd*-like genes that have been identified: *nrd*A, B, C, C.11, D, G, and H. The closest one for KVP40.0146 HE was nrdC.11 (KVP40.0153; 88930.89970), but it was still too distant to be a neighbor of KVP40.0146 to form a good setting as the T4 *mob*C/D/E. Similarly, four *nrd* genes were found in *Vibrio cholerae* ICP1 but without any HE insertion. Therefore, KVP40 and ICP1 did not have the same organization of T4 HEs.

KVP40, sharing the same host as ϕpp2, has only one putative *seg*C/D-type KVP40.0146 (complement 85073.85768), which was also similar in part to T4 *seg*B/E and *I-Tev*III, even *nrd*B.1 [9]. Therefore, the two giant *Vibrio* phages could partially cross the boundary line at *nrd*B.1 (Figure 4A), in the same host of *V. parahaemolyticus*, to catch the genes and evolve for the future form as the Enterobacteria phage T4 did. The mechanism for the gene exchange and/or evolution may also be similar to the PEG79, PEG119, and PEG 274 in the ϕpp2 as mentioned above.

## Conclusions

In summary, the phage ϕpp2 was characterized by the morphology, growth, and genomics. In the complete genome sequence analysis in this study, three giant indels and the *mob*E-type HE signified the *Vibrio* phage ϕpp2 to be a new species of T4-related phages, different from KVP40. Our analysis suggested that ϕpp2 PEG274 was an active *mob*E gene with transcriptional and translational elements. In the same host, *Vibrio parahaemolyticus*, the new phage ϕpp2 can complement its *mob*-type HE functions with KVP40 that only carries a *seg*-type HE gene. This spectrum of genome datasets of T4-related *Vibrio* phages that can co-infect the same host will be useful to investigate the hypothesis that a lateral transfer of freestanding HEs with self-mobility may result in genomic mosaicism by recombining a variety of genetic sequences in phage genomes [18].

## Competing interests

The authors declare that they have no competing interests.

## Authors’ contributions

CSL conceived and designed the study. YRL and CSL did the experiments, analyzed the sequence and wrote the manuscript. All authors read and approved the final manuscript.

## Supplementary Material

Additional file 1 **Table S1.** The list of genes with low similarity between phages KVP40 and ϕpp2. Click here for file

Additional file 2 **Text S2.***Mob*-like gene searches in detail. Click here for file
