# Supplementary material for: Genome-wide characterization of vibrio phage ϕpp2 with unique arrangements of the mob-like genes
Source: BMC Genomics. 2012 Jun 7;13:224. doi: 10.1186/1471-2164-13-224 (PMC3468402; doi:10.1186/1471-2164-13-224)
Supplement: Additional file 1 — Table S1. The list of genes with low similarity between phages KVP40 and ϕpp2. [file 1471-2164-13-224-S1.pdf]

SUPPLEMENTs

Genome-wide characterization of *Vibrio* phage ϕpp2 with unique arrangements of the *mob*-like genes

Ying-Rong Lin and Chan-Shing Lin*

Department of Marine Biotechnology and Resources, Asia-Pacific Ocean Research Center, National Sun Yat-sen University, Kaohsiung 80424, Taiwan

* For correspondence. E-mail: shinlin@faculty.nsysu.edu.tw; Tel. (+886) (0)7-525-2000 ext. 5035; Fax. (+886) (0)7-5255020

Supplement 1 (Table): **The list of genes with low similarity between phages KVP40 and ϕpp2**.

Supplement 2. The list of genes with low similarity between phages KVP40 and ϕpp2

A. Low similarity

KVP40 Gene ID	Length	ϕpp2 Gene ID	Similarity %	Function
fig|75320.1.peg.89	78	fig|75320.3.peg.88	93.51	T4-like phage PseT
fig|75320.1.peg.145	232	fig|75320.3.peg.79	25.86	MobE homing endonuclease
fig|75320.1.peg.169	112	fig|75320.3.peg.167	84.93	hypothetical protein
fig|75320.1.peg.175	130	fig|75320.3.peg.174	93.02	hypothetical protein
fig|75320.1.peg.201	94	fig|75320.3.peg.199	76.34	hypothetical protein
fig|75320.1.peg.202	90	fig|75320.3.peg.200	68.54	hypothetical protein
fig|75320.1.peg.206	83	fig|75320.3.peg.205	79.17	hypothetical protein
fig|75320.1.peg.229	60	fig|75320.3.peg.228	93.22	hypothetical protein
fig|75320.1.peg.237	205	fig|75320.3.peg.235	39.09	hypothetical protein
fig|75320.1.peg.240	49	fig|75320.3.peg.237	85.11	hypothetical protein
fig|75320.1.peg.261	79	fig|75320.3.peg.258	93.59	hypothetical protein
fig|75320.1.peg.306	77	fig|75320.3.peg.306	78.38	hypothetical protein
fig|75320.1.peg.309	135	fig|75320.3.peg.308	84.8	hypothetical protein
fig|75320.1.peg.370	209	fig|75320.3.peg.372	78.37	hypothetical protein
fig|75320.1.peg.371	103	fig|75320.3.peg.373	86.81	hypothetical protein

B. Phage ϕpp2 genes that did not match to KVP40

ϕpp2 Gene ID	start	stop	Length	Function
fig|75320.3.peg.54	30104	30322	219	hypothetical protein
fig|75320.3.peg.111	67208	67474	267	hypothetical protein
fig|75320.3.peg.119	72615	71914	702	Phage-associated homing endonuclease
fig|75320.3.peg.134	80741	81268	528	hypothetical protein
fig|75320.3.peg.144	86694	87380	687	hypothetical potassium channel protein
fig|75320.3.peg.164	97532	97705	174	hypothetical protein
fig|75320.3.peg.184	104679	104825	147	hypothetical protein
fig|75320.3.peg.168	98796	99335	540	hypothetical protein
fig|75320.3.peg.169	99731	99853	123	hypothetical protein
fig|75320.3.peg.173	101293	101466	174	hypothetical protein
fig|75320.3.peg.204	112469	112693	225	hypothetical protein
fig|75320.3.peg.259	137477	138619	1143	hypothetical protein
fig|75320.3.peg.274	149293	149964	672	Phage-associated homing endonuclease
fig|75320.3.peg.296	165535	162206	3330	Phage tail fibers; match to # 297
fig|75320.3.peg.312	177108	176899	210	Aeromonas phages 44RRORF128c [44RR2.8t] or 65p288 [Ae65]
fig|75320.3.peg.314	178980	178531	450	hypothetical protein
fig|75320.3.peg.315	179603	179731	129	hypothetical protein
fig|75320.3.peg.316	180614	180501	114	hypothetical protein
fig|75320.3.peg.320	182658	182290	369	hypothetical protein
fig|75320.3.peg.330	190507	190094	414	T4 gp2 DNA end protector protein during packaging
fig|75320.3.peg.364	228910	227813	1098	tRNA nucleotidyltransferase in Bacteria

C. KVP40 genes that did not match to phage ϕpp2

KVP40 Gene ID	start	stop	Length	Function
fig|75320.1.peg.33	19279	19485	207	Phage protein
fig|75320.1.peg.44	24009	24191	183	Phage protein
fig|75320.1.peg.52	28079	28267	189	Phage protein
fig|75320.1.peg.93	57188	57358	171	Phage protein
fig|75320.1.peg.102	61372	61992	621	Phage protein
fig|75320.1.peg.144	84923	85078	156	Phage protein
fig|75320.1.peg.150	88652	88933	282	Phage protein
fig|75320.1.peg.170	98546	98713	168	Phage protein
fig|75320.1.peg.174	100628	100981	354	Phage protein
fig|75320.1.peg.192	106833	107126	294	Phage protein
fig|75320.1.peg.200	110510	110620	111	Phage protein
fig|75320.1.peg.239	125812	125937	126	Phage protein
fig|75320.1.peg.307	172144	172413	270	Phage protein
fig|75320.1.peg.314	178767	178931	165	Phage protein
fig|75320.1.peg.318	181752	181531	222	Phage protein
fig|75320.1.peg.319	182039	182263	225	Phage protein
fig|75320.1.peg.367	231310	231191	120	Phage protein
