# Supplementary material for: Genome-wide characterization of vibrio phage ϕpp2 with unique arrangements of the mob-like genes
Source: BMC Genomics. 2012 Jun 7;13:224. doi: 10.1186/1471-2164-13-224 (PMC3468402; doi:10.1186/1471-2164-13-224)
Supplement: Additional file 2 — Text S2.Mob-like gene searches in detail. [file 1471-2164-13-224-S2.docx]

SUPPLEMENTs

Genome-wide characterization of *Vibrio* phage φpp2 with unique arrangements of the *mob*-like genes

Ying-Rong Lin and Chan-Shing Lin*

Department of Marine Biotechnology and Resources, Asia-Pacific Ocean Research Center, National Sun Yat-sen University, Kaohsiung 80424, Taiwan

* For correspondence. E-mail: [shinlin@faculty.nsysu.edu.tw](mailto:shinlin@faculty.nsysu.edu.tw); Tel. (+886) (0)7-525- 2000 ext. 5035; Fax. (+886) (0)7-5255020

Supplement 2:

**Mob-like Gene Searches in Detail:**

In whole genome searches to find the matched pairs of target gene with its flanking neighbors in order to classify the *mob*/*seg* types, we encountered a complexity of combinations in which not only the gene mobility alter the relationship between the target and its neighbors, but also the one target and two flanking-neighbor genes evolved independently by mutagenesis. In addition to pair-wise comparison for individual gene to find the high similarity, we also implemented a twofold strategy. First (the neighbor-direct method), the neighbors of *mob*/*seg* genes in φpp2 were PSI-BLAST searched within other T4-like phage genomes and the potential functions of such φpp2 *mob* neighbors were assigned for further comparison of the uniqueness to 15 types of HE and their neighbors. The phage T4 neighbors can also be directly compared with the φpp2-HE neighbors. The neighbor-direct method is the simplest way to find a co-evolution of the target gene with its neighbors. This will reveal the insertion/deletion of the target gene during the evolution if the upstream gene is well paired with the downstream genes in the genomes. Second (the neighbor-indirect method), a φpp2-HE candidate is projected to one locus in the known phage T4 genome; the T4-corresponding neighbors thereafter are pair-wise aligned with whole φpp2 genome. This neighbor-indirect method guarantees that the functions of neighbors remain in good conditions of HE settings. The new back-projected neighbors may provide the information about a suitable slot for the *mob*/*seg* genes of interest to go to or to come from; the difference is due to the vision of evolution that comes from the past or goes to the future of the gene mobility. The paradigm can be applied to other pairs of genomes or gene sets to be compared with.

The Mob types of φpp2 were identified according to the orientation similarity to the neighbor ORFs of 15 homing endonucleases in Enterobacteria phage T4, in which *mob*, *seg*, and *I-Tev* were located in the genome map with unique flanking arrangements. *mob*B is a neighbor to α.gt (glucosyl transferase) in phage T4 while the functions of *mob*A neighbors has not yet been identified. To identify the neighbors of *mob* genes in φpp2, the α.gt and *nrd* (ribonucleotide reductase) orthologs were searched around the genome. No match to T4 α.gt or β.gt was found in entire genomes of φpp2 and KVP40 (NC_005083); therefore, the *mob*B-like gene could not exist in φpp2.

The neighboring genes, which can be used to help classify the variants of subtypes, for the *mob*C, *mob*D, and *mob*E are flanked by *nrd*D/*nrd*G, one *nrd*C.11, and *nrd*A/(*I-Tev*III)/*nrd*B, respectively. In addition, the arrangement of the *mob*D promoter differed from that of *mob*C/E. Three *nrd*-like genes were found in φpp2: one was found explicitly by the RAST and three others were implicit but manually confirmed with PSI-BLAST searches. Similar to the settings of *nrd*D/*nrd*G and *nrd*A/*nrd*B pairs in phage T4, the implicit pair of PEG12 (612 aa; 7583..9418, 1836 nt) and PEG15 (159 aa, 10506..10982; 477 nt) was found in φpp2. The PEG12 protein was similar to the large subunit of anaerobic ribonucleotide reductase of class III (EC 1.17.4.2), with 52.05% similarity to T4 *nrd*G, while PEG15 was assumed to be the activating protein (EC 1.97.1.4) for the ribonucleotide reductase with 52.74% similarity to T4 *nrd*D. PEG132 matched to T4p232 (*nrd*B.1, complement 139716..139967), which denoted as *nrd*B.1 in the boundary of MobE and downstream close by *seg*D. For the fourth *nrd*-like, 1041 nt of PEG148 (347 aa, 89176..90216) in φpp2 was mapped to T4 nrdC.11.

Using the neighbor-indirect method to map the *mob*C, φpp2 PEG274 (149293..149964, 672nt) was first matched to T4p075 (*mob*C, complement 43538..42906, 633 nt). The neighbor gene T4p074 (nrdG, complement 42446..42916, 471 nt) was back-projected to φpp2 PEG15 (10506-10982, 477nt) with the similarity of 52.05%; while, another neighbor gene T4p076 (nrdD, complement 43535..46385, 3171 nt) was matched to φpp2 PEG12 (7583-9418, 1836 nt) with a similarity of 52.74%. The distance of the PEG12/15 pair from proposed PEG274 was at least 104040 nt apart, although the pair of PEG12 and PEG15 seems to be a good site for an HE to situate.

In the locus integration of Mob genes and their aforementioned neighbors to classify the types of H-N-H homing enzymes, none of the φpp2 PEG79 (49482..48856, 627 nt), PEG119 (72615..71914, 702 nt) and PEG274 (149293..149964, 672 nt) was qualified to be *mob*B, *mob*C, *mob*D, or *mob*E. To qualify PEG79, the PEG12/15 was 37,874 nt apart, and PEG148 (8917..90216, 1041 nt) was in unreachable distance of 39694. To qualify PEG119, the PEG12/15 was too far apart with 60932 bp, and PEG148 was at a distance of 16561 bases. To qualify PEG274, either 104040 nt to PEG12 or 138311 nt to PEG15 was farther remote, and PEG148 was still too far for the neighbor adjunction within the intergenic space of 59077 nt.

Alternatively, using the neighbor genes just around the three candidates of homing endonucleases (the so-called neighbor-direct method), the neighbors of φpp2 PEG79, PEG119, and PEG274 were *de novo* manually searched with PSI-BLAST. Neither neighbors of PEG79 (peg70-peg78 and PEG80-peg90) nor PEG119 (peg110-peg117 and PEG 120-peg125) were in any way close to *nrd*-like genes.

Broadening the search range to examine φpp2-peg119 for possibly being a *mob*D-like – needing only a single side of *nrd*-like gene, two closer *nrd-*like candidates were PEG132 (79809..80003, 195 nt) and PEG148 (89176..90216, 1041 nt), which aligned to T4p095 (*nrd*C.11, complement 55435..56445) with 29.48% match but the location was too distant. A different neighbor φpp2 PEG132 was denoted as *nrd*B.1, which was matched to T4p232 (*nrd*B.1, complement 139716..139967) in the boundary of MobE and downstream close by *seg*D. However, PSI-BLAST did not confirm this role; a part of 60 aa in the φpp2 PEG132 additionally 48% matched to phospho-N-acetylmuramoyl-pentapeptide-transferase of *Aeromicrobium marinum* DSM 15272. The φpp2 PEG119 could be considered to be a different proto-type of a homing endonuclease, surrounding which the neighbors were inserted to T4 MobD/E settings.

In *de novo* identification of a *mob*-type for φpp2 PEG274 (149293..149964, 672 nt) using the neighbor-direct method, φpp2 PEG273 (147028..149253, 2226 nt) of the upstream neighbor gene was blasted to NrdA of *Aeromonas* phages (PX29, phiAS5), Enterobacteria phages (JSE, RB49, phi1, T4) and *Shigella* phage SP18. The downstream neighbor PEG275 (149957..151081, 1125 nt) was blasted to NrdB of *Aeromonas* phages phiAS5, Aeh1, *Klebsiella* phage KP15, and Enterobacteria phage RB16. Another neighbor, PEG276 (151083-151382, 300 nt), was also blasted to the NrdC thioredoxin; it aligned well as 86% homologous to NrdC thioredoxin in *Aeromonas* phages phiAS5, Aeh1, and 65, as well as to *Klebsiella* phage KP15, *Shigella* phage SP18, and Enterobateria phages RB16, RB43 and ime09. With the matches of upstream and downstream of *nrd*-like genes which complemented the full structure of MobE neighbors, the φpp2 PEG274 can be annotated as MobE-type HE, without the existence of *I-Tev*III intron yet.

Similarly, KVP40.0146 (complement 85073..85768 in NC_005083, 696 nt) encodes 231 aa, which was PSI-BLAST to GIY-YIG endonuclease genes, including *Aeromonas* phages (phage 25 and phiAS5), *Acinetobacter* phages (Acj61 and Ac42), Chlorella virus FR483, Enterobacteria phages (RB51, RB16, and T4), *Klebsiella* phage KP15, and *Staphylococcus* phage PH15. As shown in Fig. 5A, the phylogenetic analysis plotted KVP40.0146 to be a *seg*C/D type.

Using the neighbor-direct method, KVP40.0145 (84923-85078, 156 nt) and KVP40.0147 (85926-86240, 315 nt) could not match to any protein of known function (Fig. 6D). As Table 3 shows, the homologs for KVP40.0146 were blasted to *seg*A/C/D/E and *I-Tev*I, as well as an upstream of MobE (It is *nrd*B.1 similar to φpp2 PEG132.). Using the neighbor-indirect method, T4 *seg*D ([NP_049788.2](http://www.ncbi.nlm.nih.gov/protein/29366681?report=genbank&log$=prottop&blast_rank=41&RID=61TTWVNH014)) and *seg*E (NP_049795) were flanked by characteristic genes of gp23/24 and *inh*/*uvs*W, respectively. The back-projected genes in KVP40 for gp23/24 were KVP40.0363 (gp23, 224506..226050, 1545 nt), matching to phage major capsid protein of Caudovirales, and KVP40.0063 (gp24, 36306-37202, 897 nt) as the phage capsid vertex protein. Those back-projection genes for *inh*/*uvs*W were KVP40.0367 (*inh*, 229118-229609, 492 nt), encoding inhibitor of prohead protease gp21, and KVP40.0378 (*uvs*W, 235320-236843, 1524 nt) for DNA helicase. Both were too distant to bracket the KVP40.0146 of GIY-YIG endonuclease gene.

Using the neighbor genes of T4 HEs to recognize the potential loci for the homing endonucleases, types of *mob*C, *mob*D, and *mob*E can be classified by neighbor elements as well the different arrangements of their promoters: nrdD-mobC-nrdG, mobD-nrdC.11, and nrdA-(I-TevIII)-mobE- nrdB, respectively. In KVP40, there are seven *nrd*-like genes that have been identified: *nrd*A, B, C, C.11, D, G, and H. The closer one for KVP40.0146 HE was nrdC.11 (KVP40.0153; 88930..89970), but it was still too distant to be a neighbor of KVP40.0146 to form a good setting as the T4 *mob*C/D/E.

KVP40, sharing the same host as φpp2, owns only one putative *seg*C/D-type KVP40.0146 (complement 85073..85768), which was also similar in part to T4 *seg*B/E and *I-Tev*III, even *nrd*B.1 [9]. Therefore, the two giant *Vibrio* phages could partially cross the boundary line at *nrd*B.1 (Fig. 5A), in the same host of *V. parahaemolyticus*, to catch-the-fly and evolve for the future form like the Enterobacteria phage T4 did. The mechanism for the gene exchange and/or evolution may also be similar to the PEG79, PEG119 and PEG 274 in the φpp2 as mentioned in the manuscript.

The neighbor-direct method provides straightforward results when co-evolution of the target and neighbor genes exists. Nevertheless, the neighbor-indirect method provides a wide range of searches for the potential lots of HE neighbor genes, which the mob had evolved from or was evolving towards. The φpp2 PEG79 and PEG119 therefore were re-located into being neighbors of the PEG274 because they linked downstream of PEG273 (Table 3). The *mob*E was also identified as a good suit for the PEG156 in *Aeromonas* phages 65 and Aeh1. Additionally, Table 3 includes several consistent pairs of neighboring genes which may be good candidates for future investigations. The *mob*C-(*I-Tev*I) was flanked well by Aeh1 PEG41/42, phage 65 PEG52/53, KVP40 and φpp2 PEG12/15. The *seg*D neighbors were Aeh1 PEG235/236 and P-SSM2 PEG136 (split). The *seg*G was flanked by phage 65 PEG81/82, KVP40/φpp2 PEG4/5, and P-SSM2 PEG7/9.
